# Supplementary material for: Combined inhibition of SHP2 overcomes adaptive resistance to type 1 BRAF inhibitors in BRAF V600E-driven high-grade glioma
Source: Neurooncol Adv. 2025 Aug 2;7(1):vdaf170. doi: 10.1093/noajnl/vdaf170 (PMC12400027; doi:10.1093/noajnl/vdaf170)
Supplement: vdaf170_suppl_Supplementary_Materials_1 [file vdaf170_suppl_supplementary_materials_1.docx]

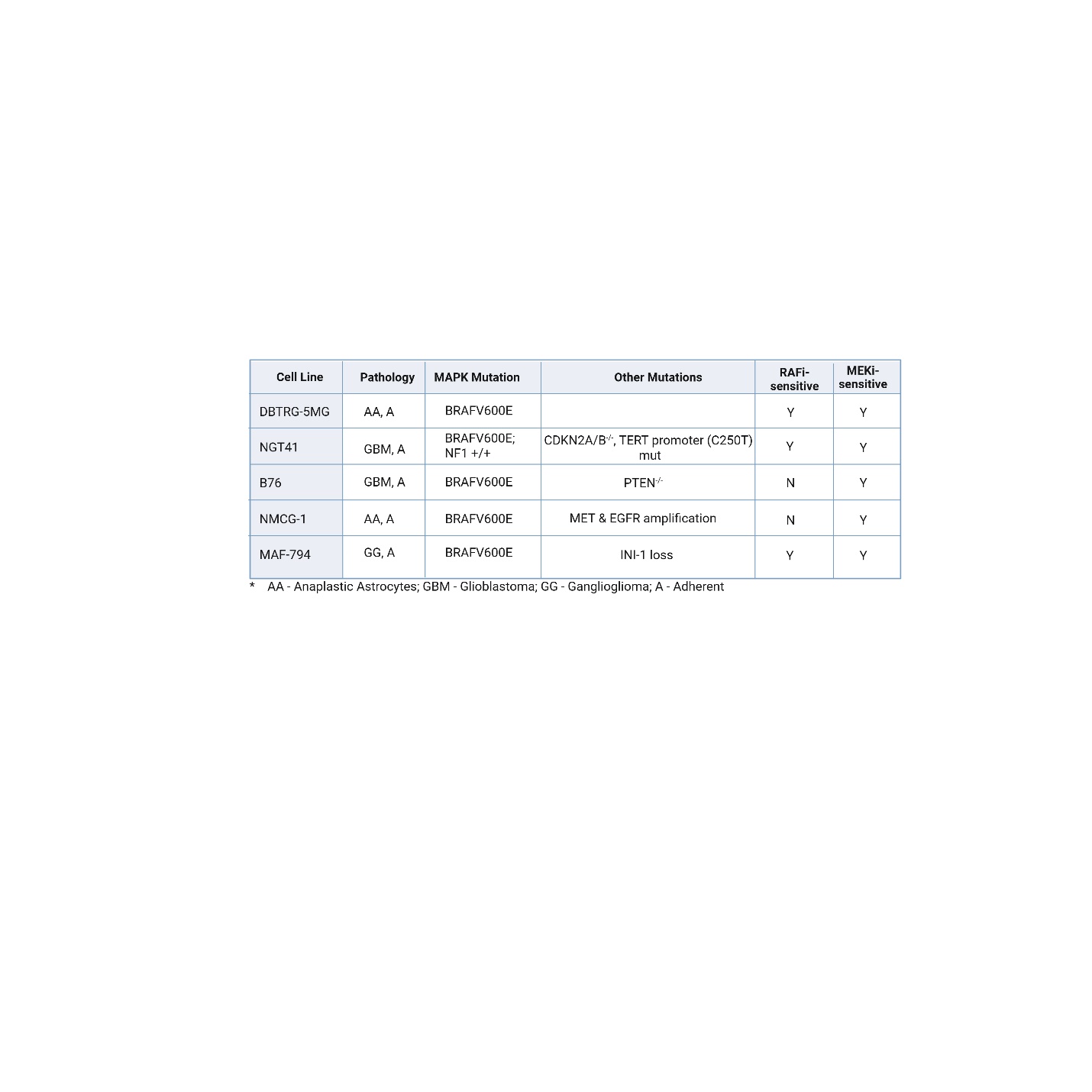


**Supplementary Table 1.** Cell lines used in this study, including their pathology, MAPK mutation, co-occurring mutations, and sensitivity to RAF or MEK inhibition.


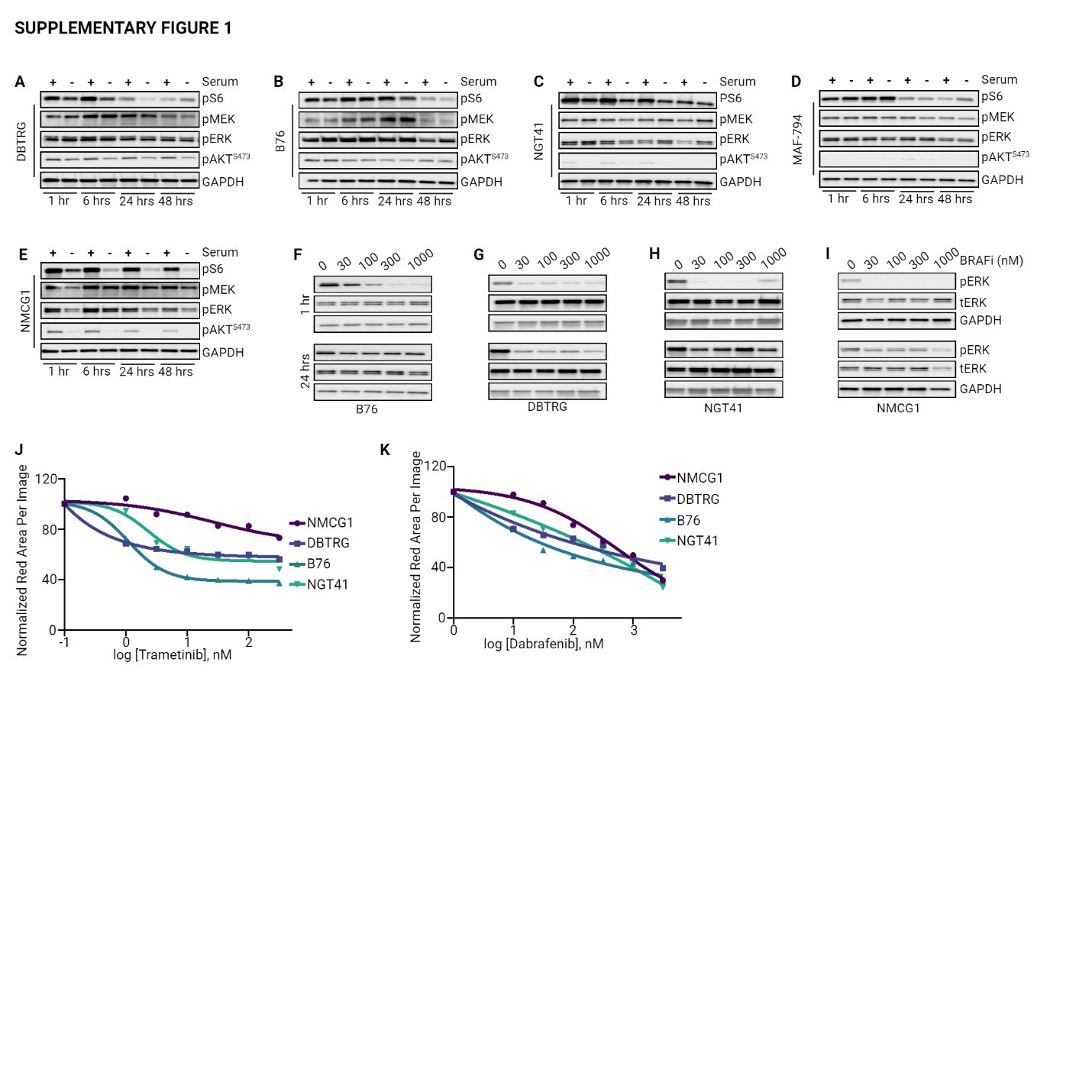


**Supplementary Figure 1:** Administration of 10-15% fetal bovine serum compared with serum starvation over time in A) DBTRG, B) B76, C) NGT41, D) MAF-794, and E) NMCG1 glioma cell over time. F) Immunoblots from cells treated with increasing doses of dabrafenib (30nM-1000nM) showing ERK signaling at 1 and 24 hours in B76, G) DBTRG, H) NGT41, I) and NMCG1 cells. Dose-response curves to J) trametinib or K) dabrafenib monotherapy in BRAF V600E mutant glioma cells treated with increasing drug doses for five days.


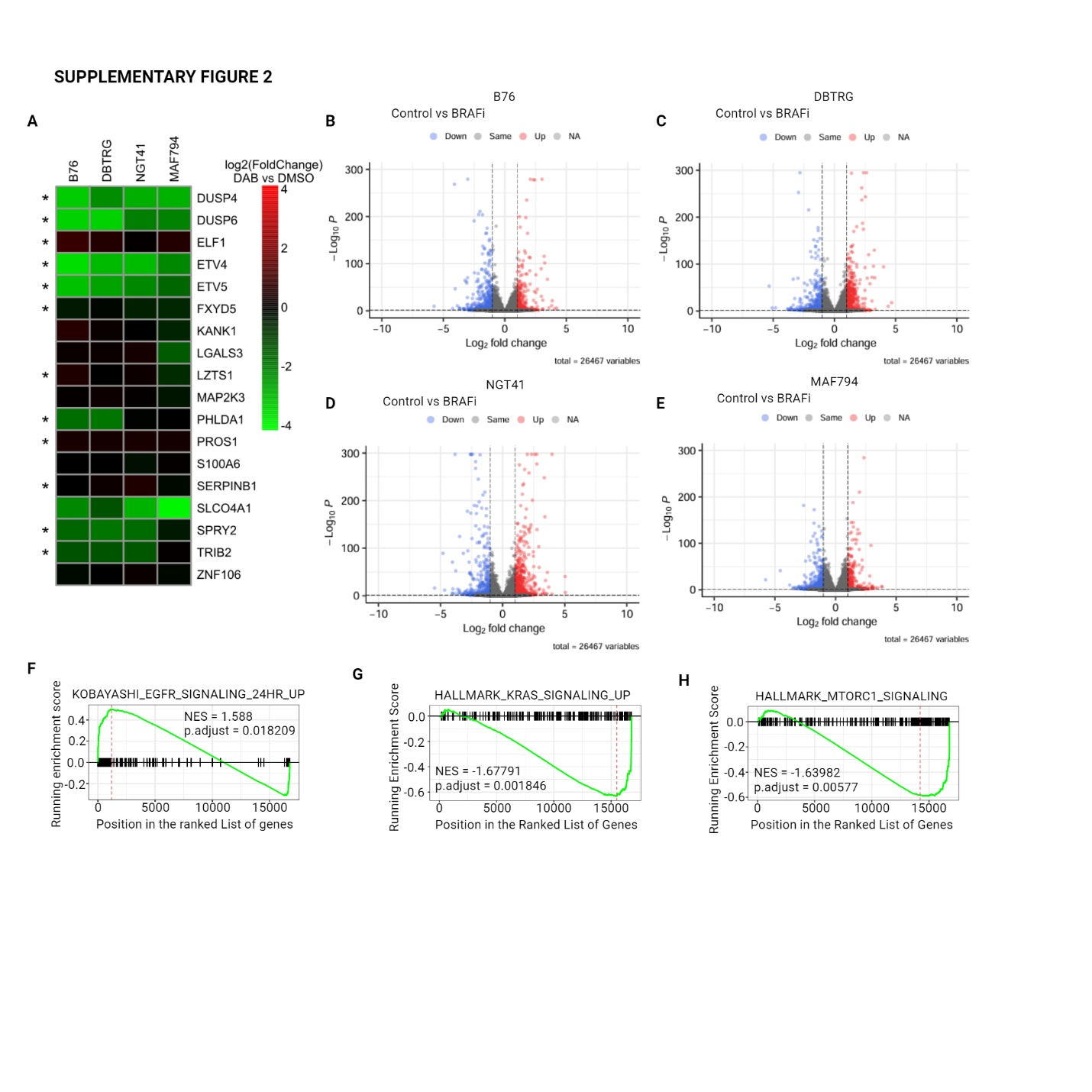


**Supplementary Figure 2:** A) Expression of an 18-gene signature for tumor-independent MEK functional output showing log2FC with respective p values of transcripts in four cell lines treated with BRAFi vs control. Volcano plots illustrating positive and negative LFC (log fold change) transcripts in BRAFi-treated (dabrafenib, 100nM)-treated B) B76, C) DBTRG, D) NGT41), and E) MAF794 cells compared to controls. F-H) Barcode plots of selected significantly altered pathways relevant for MAPK signaling following BRAFi compared to vehicle.


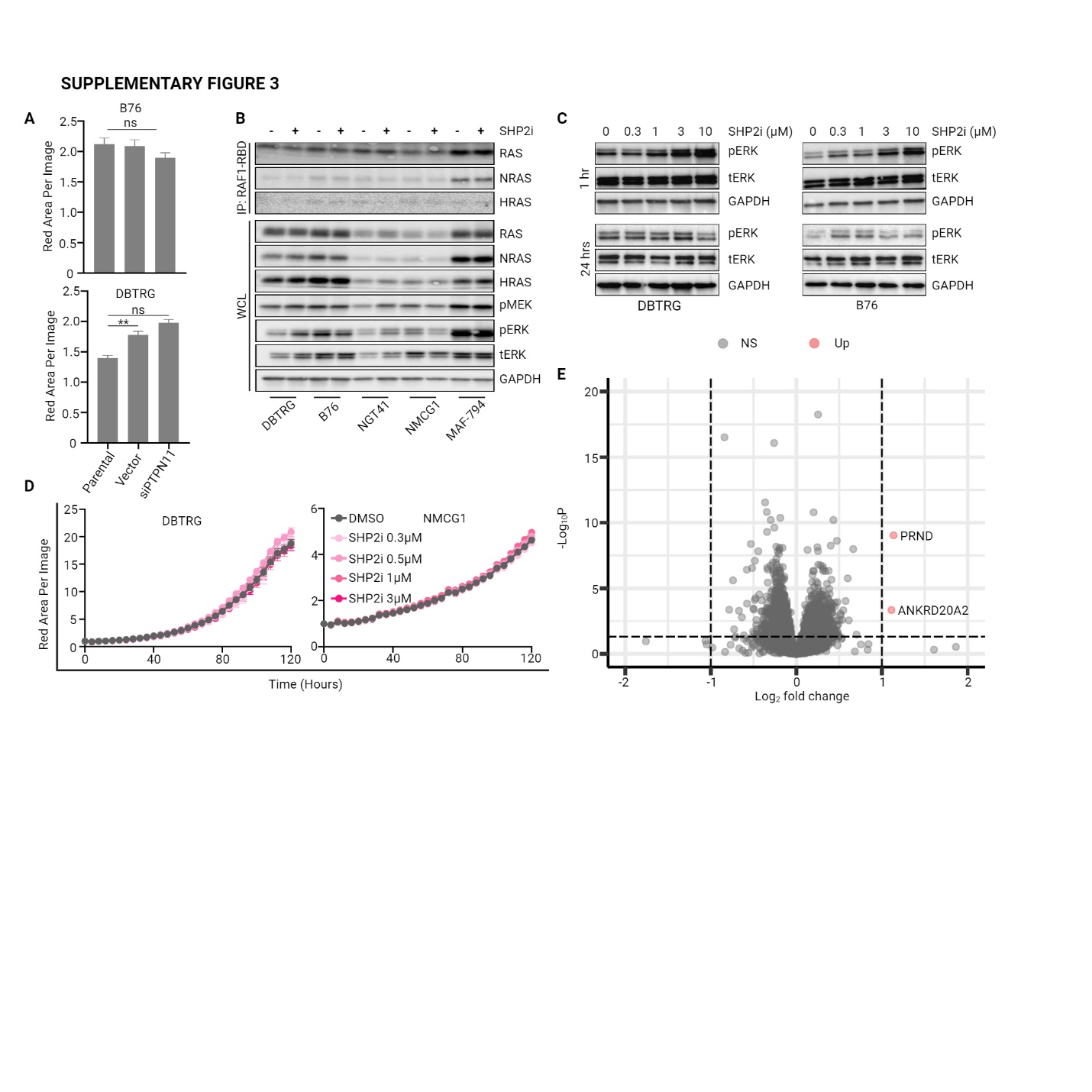


**Supplementary Figure 3:** A) Relative cell number measured by a red nuclear label (dsRed) 4.5 days after transduction with either vector or siPTPN11. B) RAS-GTP pulldown assay 24 hours after TNO155 treatment (3 µM); WCL = whole cell lysate. C) Two BRAF V600E mutant glioma lines (DBTRG and B76) were treated with increasing doses of TNO155 (0.3 µM-3 µM) at 1 hour and 24 hours after treatment. D) Cell growth over time following treatment with increasing doses of TNO155 (0.3-3 µM), normalized to baseline cell density per image as quantified by red nuclear area. E) Volcano plot of differentially expressed transcripts in response to TNO155 (3 µM) monotherapy averaged across 4 cell lines.


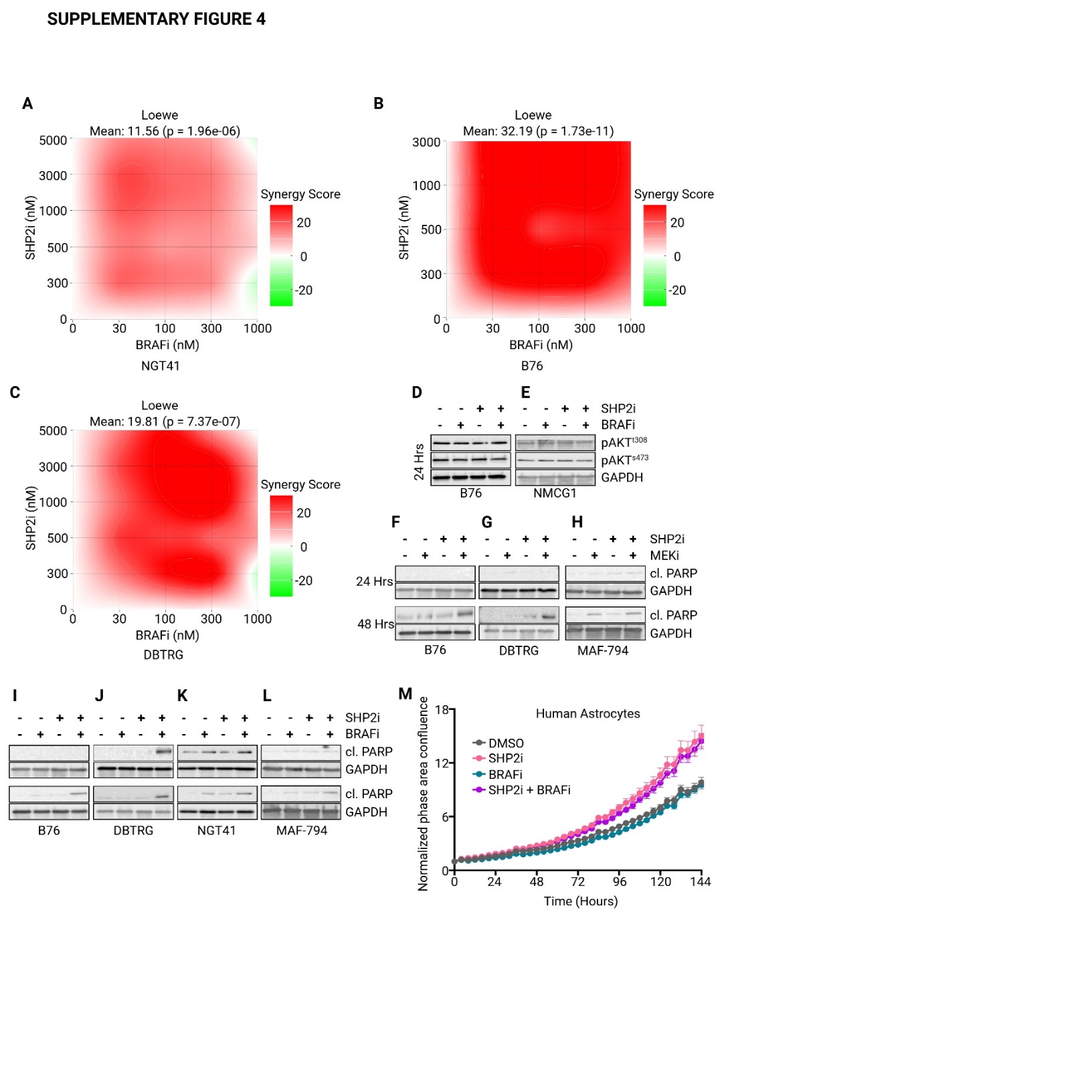


**Supplementary Figure 4:** A-C) Loewe’s synergy heatmaps of TNO155 combined with dabrafenib in NGT41, B76, and DBTRG cells. D-E) Immunoblots of phospho-AKT 24 hours after treatment with BRAFi, SHP2i or the combination. F-H) Immunoblots of cleaved PARP expression in B76, DBTRG, and MAF-794 lines at 24 and 48 hours after treatment with MEKi (30nM, trametinib), SHP2i (3 μM, TNO155) or the combination. Samples are from the same experiment as main Figure 3C. I-L) Immunoblots of cleaved PARP expression in B76, DBTRG, NGT41, and MAF-794 lines at 24 and 48 hours after treatment with BRAFi (100 nM, dabrafenib), SHP2i (3 μM, TNO155), or the combinations. Samples are from same as experiment as main Figures 4E or 6J. <) Growth of human astrocytes measured with Incucyte live cell-imager after treatment with dabrafenib (100 nM), TNO155 (3 μM), or the combination.


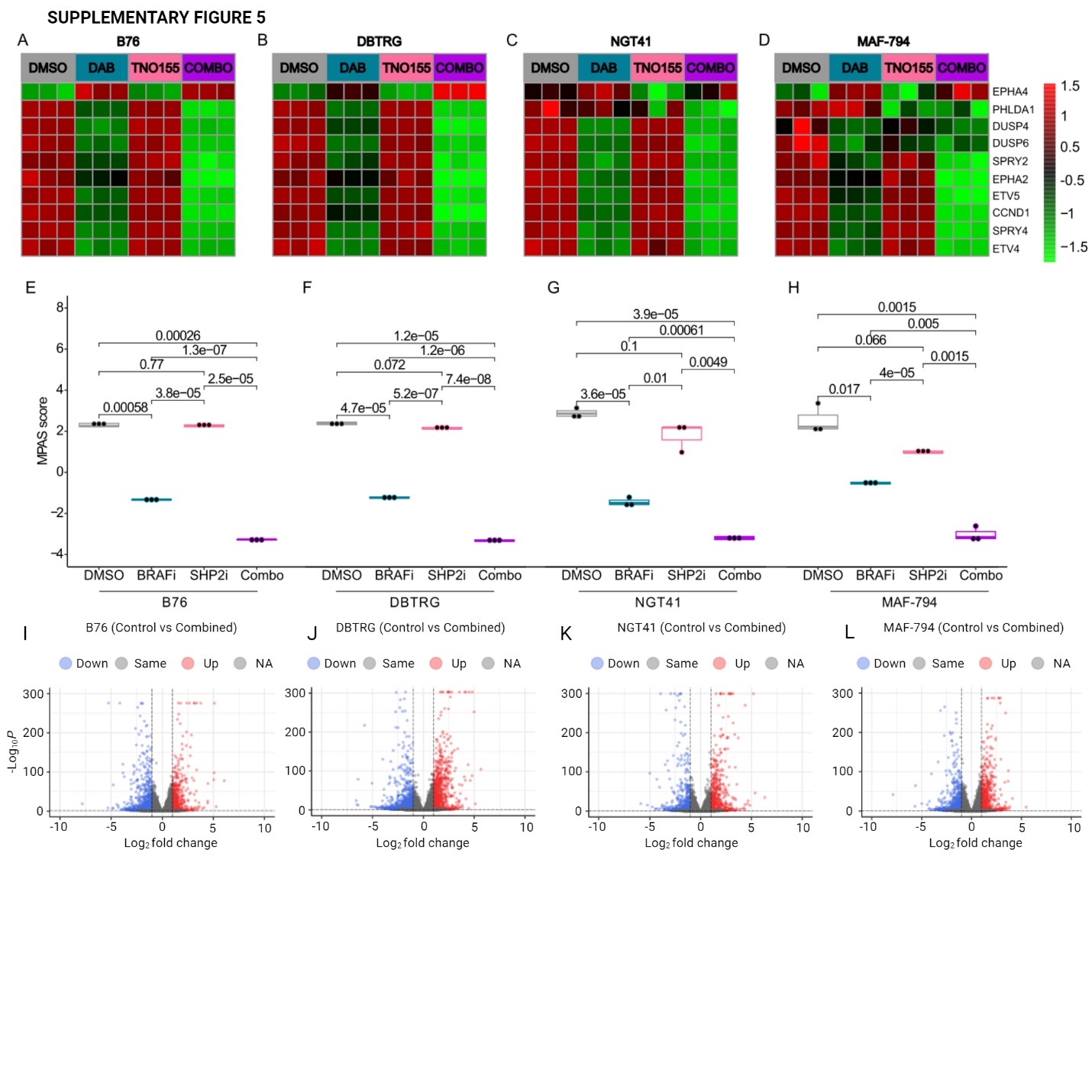


**Supplementary Figure 5:** Heatmap of log2 TPM of the 10 genes in the MPAS signature in triplicates for A) B76, B) DBTRG, C) NGT41 and, D) MAF-794 cells treated with DMSO, dabrafenib, TNO155, or the combination. E-H) Box-and-whisker plots displaying mean scaled expression of MPAS score for B76, DBTRG, NGT41, and MAF794 cell lines treated with DMSO, dabrafenib, TNO155, or combination. I-L) Volcano plots showing positive and negative LFC (log fold change) transcripts in BRAFi + SHP2i (dabrafenib-100 nM, TNO155-3 μM)-treated A) B76, B) DBTRG, C) NGT41), and D) MAF794 as compared to controls.


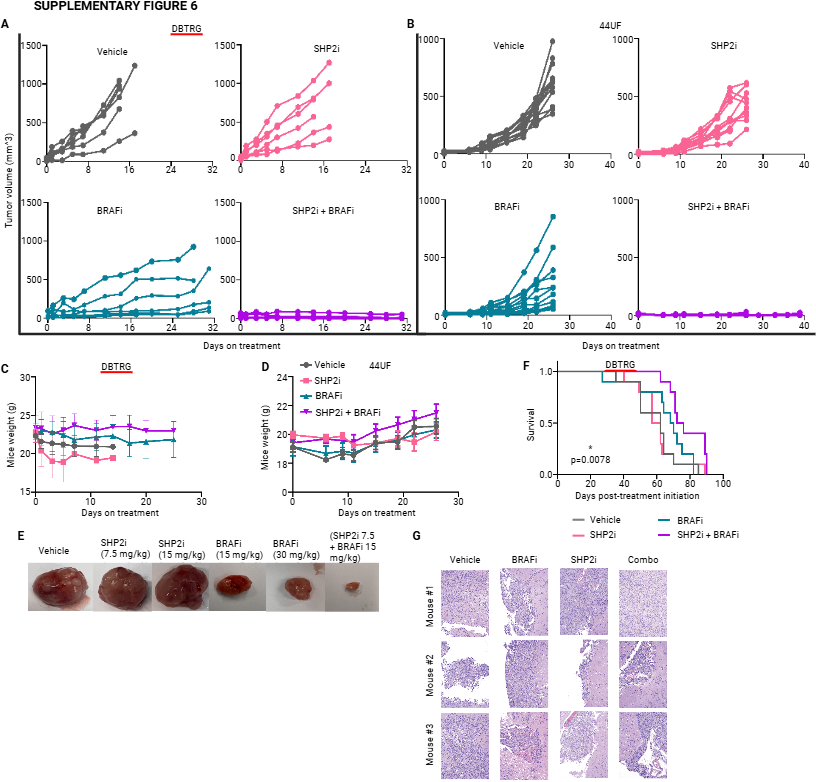


**Supplementary Figure 6:** A/B) spider plot of individual tumor volume in mice flanks for DBTRG and 44UF experiments. C/D). Mice weight over time on treatment for each study. E) Tumor size at end of study for higher monotherapy doses of TNO155 and dabrafenib in NGT41 xenografts. F/G) Orthotopic, intracranially-implanted DBTRG xenografts were treated as above for 4 weeks starting from day 14 post-implantation. Survival was recorded by an independent, blinded evaluator. Hazard Ratio (log rank) of the combination versus dabrafenib was 2.172. Error bars represent mean ± SEM (*p ≤0.05). Brains were formalin fixed, embedded, and stained. Tumors were evaluated and photographed by a neuropathologist for visual comparison.
